# Specialty grand challenge: Rehabilitation engineering

**DOI:** 10.3389/fresc.2023.1069269

**Published:** 2023-02-22

**Authors:** Ping Zhou

**Affiliations:** School of Rehabilitation Science and Engineering, University of Health and Rehabilitation Sciences, Qingdao, China

**Keywords:** rehabilitation engineering, collaboration, patient need, science, education

## Introduction

Rehabilitation engineering is the use of engineering sciences and principles to provide technological solutions to improve the quality of life for people experiencing disabilities. Although rehabilitation engineering is a relatively new field of study compared with traditional engineering disciplines, earliest forms of practice date back thousands of years (1, 2). Thus, early records reveal use of rudimentary aids: walking sticks to aid ambulation, corrective lens for improved vision, and audiphones to aid hearing. The modern era of rehabilitation engineering began in the 1970 s, largely benefiting from United States government advocacy and support. In particular, the establishment of Rehabilitation Engineering Research Centers (RERC) through the National Institute on Disability, Independent Living, and Rehabilitation Research (NIDILRR), part of Administration for Community Living (formerly through National Institute on Disability and Rehabilitation Research, the United States Department of Education) has noticeably promoted development and research in rehabilitation engineering. The first rehabilitation engineering society, the Rehabilitation Engineering Society of North America (RESANA), was launched in 1979. This was followed by other national or regional professional associations in rehabilitation engineering around the world.

According to World Health Organization (WHO), 1 in 6 people worldwide currently experience a significant disability, 1 in 3 people are living with a health condition that can benefit from rehabilitation, and these numbers are expected to increase with growing aging populations and people living with chronic disease (3, 4). As rehabilitation becomes a priority health strategy for the 21st century, development of rehabilitation engineering plays a crucial role to fulfill the profound unmet need for rehabilitation worldwide. The development status of rehabilitation engineering varies across different countries, and people in low income countries have not benefited from technical advances as much as those in higher income countries. The Global Cooperation on Assistive Technology (GATE) initiative was established by WHO in 2014 to improve access to high-quality affordable assistive products globally (5). Although the past half century has witnessed a tremendous progress in rehabilitation engineering, contributing to a more supportive environment for people experiencing disabilities, there is still much to be done to meet the world's increasing needs in both advancement and use of rehabilitation and assistive technologies. In the following, we outline several grand challenges for the modern burgeoning field of rehabilitation engineering.

## Grand challenge in integrating emerging technology advances in rehabilitation engineering

Life changing breakthroughs have been made in rehabilitation engineering, resulting in a great variety of devices, tools, and systems that meet a wide range of needs for individuals experiencing functional deficits in mobility, communication, hearing, vision, or cognition (6). The achievement of rehabilitation engineering at present would not have been possible without advances in many different areas such as portable and powerful computational resources, computer-aided design and manufacture, wearable or implantable sensors, mobile and wireless technology, rapid prototyping and 3D printing, exoskeleton and robotics, neuromuscular and brain stimulation, virtual reality, brain computer interfaces, etc. (see review articles (7–15), among others). The multidisciplinary nature of rehabilitation engineering is evident that the field is inherently collaborative. Breakthrough in one field can significantly influence or even revolutionize rehabilitation engineering technologies. Integration of knowledge and advances generated by various fields of study is critical for the further development of rehabilitation engineering. There are exciting emerging advances such as artificial intelligence and machine learning, big data technology, 5 G/6 G network, autonomous automobiles/wheelchairs, smart home/IoT, new power supplies, new materials, new surgery approaches, etc. It remains an important and challenging task to keep up-to-date on these new technologies, understand their complexities, and integrate technology advances into rehabilitation engineering, which is expected to significantly benefit people experiencing disabilities.

## Grand challenge in identifying patient or consumer priorities in rehabilitation engineering

For rehabilitation engineering, much of the activities are driven by the needs of people experiencing disabilities. Rehabilitation engineers, instead of working in isolation, should work closely with patients and clinicians, viewing them as part of the team to facilitate better outcomes. It is worth noting that the current development in rehabilitation engineering has not fully met the priority needs of patients or consumers. There exists a discrepancy between areas of research of the most cited rehabilitation engineers and consumer priorities (16). A pressing improvement for future development of rehabilitation engineering is to reinforce consumer and clinician input in order to better capture and meet the priority needs of patients or consumers. On one hand, rehabilitation engineering should focus on community-based solutions in conjunction with a global/universal design. On the other hand, customized design and development based on patient-specific needs (i.e., clinical, economic, environmental, social, geographical, health system, insurance policy, psychological, emotional, etc.) may offer the best solution. Rehabilitation and assistive devices can range from low-technology ones that are inexpensive and simple to high technology ones that are complex and expensive. In the context of patient needs, rehabilitation engineers should look for the most appropriate application of technology, not necessarily application of the most advanced technology.

## Grand challenge in strengthening science in rehabilitation engineering

In a recent review of NIDILRR's RERC activities, it was reported that 70% of the research and development staff of RERCs are in engineering fields, 23% in clinical fields, and only 7% come from basic science fields (17). Although these ratios are derived from this specific funding program, they perhaps provide an approximate picture of the personnel composition in the field of rehabilitation engineering. High involvement of engineers in rehabilitation engineering is natural since its primary goal is not science itself, but application of science for practical solutions. Despite this pragmatic emphasis, basic research that may be of potential benefit to rehabilitation engineering should not be neglected. In fact, basic research is the source of engineering and technology innovation. It is critical to strengthen research in basic science in the future development of rehabilitation engineering. New knowledge produced by basic research is rewarding and inspiring for innovative solutions in rehabilitation engineering (18–20). For example, exploration of the underlying pathophysiological mechanisms associated with a functional deficit and its response to intervention can in turn help guide or improve the rehabilitation engineering practice. Therefore, although perhaps not immediately beneficial, the involvement of more scientists and science-driven activities in the long term advocates for the future development of rehabilitation engineering.

## Grand challenge in promoting education and training in rehabilitation engineering

Rehabilitation engineering also faces a grand challenge in education and training. Given the demands of the field to produce positive outcomes for patients, there is an unmet need to establish professional qualification programs in rehabilitation engineering. Initiatives to address the challenge can include establishment of common core curriculum and specialist curriculum (either as an independent discipline or as a subfield of other disciplines, such as biomedical engineering), development of textbooks and other teaching and learning materials, summer schools, continued education, and online courses, etc. Of particular note, rehabilitation technology is to serve our patients, so there is an urgent need to develop practical technology design and implementation techniques. These may well require cross-disciplinary training of engineers by clinicians and similar training of clinicians by engineers. In addition, a systematic examination has revealed gender and geographical disparities in rehabilitation engineering (16). There is a necessity of educating and training more women in the field. For countries with limited development in rehabilitation engineering, a focus on education and training may be the most effective way to catch up. A fact that should not be ignored is that there remain barriers for people experiencing disabilities to receive a professional education. These barriers should be lowered or removed, so more people experiencing disabilities can be trained to become specialists in rehabilitation engineering, thus providing a unique and important perspective for the field.

## Conclusion

As a nascent but fast-growing field of endeavor, it can be anticipated that rehabilitation engineering will remain intensely active and vibrant. The field offers nearly unlimited potential for continued assistance of day-to-day activities related to independent living, education, employment, and recreation of people experiencing disabilities. The journey promises to be exciting and hopeful, but challenging. In line with the mission of *Frontiers in Rehabilitation Sciences* (21), the “Rehabilitation Engineering” section provides a forum for those interested in engineering technologies that contribute to rehabilitation and restoration of health and well-being in people experiencing disabilities due to disease, injury, or aging. The section aims to report recent engineering and technology advances and welcomes development of innovative concepts, designs, tools, techniques, devices, and systems that assist individuals experiencing different disabilities who have unmet needs related to mobility, sensation, communication, and cognition. The section advocates multidisciplinary collaboration, patient or consumer centered development, investigation of mechanisms and science-driven activities, as well as educational and training activities in rehabilitation engineering. Taken together, the section is expected to reflect the current state of art, science, and education in the field of rehabilitation engineering, providing a valuable forum for knowledge dissemination and exchange.
